# Identification of a TLR-Induced Four-lncRNA Signature as a Novel Prognostic Biomarker in Esophageal Carcinoma

**DOI:** 10.3389/fcell.2020.00649

**Published:** 2020-07-21

**Authors:** Jing Liu, Yanbo Wang, Yanjie Chu, Ruiling Xu, Dekai Zhang, Xinhong Wang

**Affiliations:** ^1^Department of Gastroenterology and Hepatology, The Second Affiliated Hospital of Harbin Medical University, Harbin, China; ^2^Department of Thoracic Surgery, Harbin Medical University Cancer Hospital, Harbin, China; ^3^Center for Infectious and Inflammatory Diseases, Texas A&M University, Houston, TX, United States

**Keywords:** Toll-like receptor, long non-coding RNAs, esophageal carcinoma, signature, biomarker

## Abstract

Long non-coding RNAs (lncRNAs) have emerged as key regulators of Toll-like receptor (TLR) signaling to control innate immunity, and this regulatory mechanism has recently been implicated in esophageal carcinoma (ESCA). However, a comprehensive analysis of TLR-induced lncRNAs and their roles in diagnosis and prognosis in ESCA is still lacking. In this study, we first investigated the precise relationship between lncRNA perturbations and alteration of TLR signaling by constructing the lncRNA-TLRs co-expression network involved in ESCA, and identified 357 TLR-related lncRNAs. Of them, four TLR-related lncRNAs (*AP000696.1*, *LINC00689*, *LINC00900*, and *AP000487.1*) are significantly associated with the overall survival (OS) of ESCA patients, and utilizing this four-lncRNA signature is capable of stratifying patients into high-risk and low-risk groups with significantly different OS in the discovery set. Further analysis in different independent patient sets also confirmed the robustness of the prognostic value of the four-TLR-lncRNA signature in predicting the OS of ESCA patients. Moreover, the results of multivariate analysis in different patient sets indicated that the four-TLR-lncRNA signature is an independent factor after adjusted by other clinical factors. Thus, we have identified a TLR-induced four-lncRNA signature, which represents a promising prognosis biomarker for ESCA, and our study might provide new candidate targets for therapeutic intervention via targeting TLR-induced lncRNAs in ESCA patients.

## Introduction

Esophageal carcinoma (ESCA) is a common type of malignant tumor in the digestive system. Two major pathologic subtypes of ESCA are esophageal adenocarcinoma and esophageal squamous cell carcinoma (ESCC; Napier et al., 2014; Jain and Dhingra, 2017). There were an estimated 18,440 newly diagnosed cases and 16,170 deaths of ESCA, which account for approximately 1% of all diagnosed cancer cases and 2.7% of all cancer-related deaths in the United States (Siegel et al., 2020). Although ESCA could be treated by esophagectomy in combination with chemotherapy and radiation therapy, the outcome of ESCA is generally tended to be relatively poor, and the 5-year relative survival rate is approximately 20% due to the late diagnosis (Mawhinney and Glasgow, 2012; D’Journo and Thomas, 2014; Rustgi and El-Serag, 2014; Siegel et al., 2020). TNM staging system was most commonly used for guiding clinical decision making but is still insufficient for improving ESCA diagnosis and prognosis because of intrinsically molecular heterogeneity of ESCA (The Cancer Genome Atlas Research Network, 2017; Liu et al., 2017). Therefore, molecular biomarkers were urgently needed for complementing the TNM staging system and providing more precise prediction and consequently improved personalized cancer care.

Toll-like receptors (TLRs), one class of pattern-recognition receptors (PRRs), play crucial roles in the innate immune system by recognizing pathogen-associated molecular patterns (Kawasaki and Kawai, 2014). It has been suggested that TLR-mediated inflammation promotes tumor growth and development (Cen et al., 2018). TLRs have also been found to be involved in the tumor cell death by inducing apoptosis, autophagy, and programmed necrosis in tumor cells (Cen et al., 2018). Members of the TLR family, including ten TLRs, have been discovered in humans, and it has been reported that some of them are up-regulated in many tumor cells, tissues, or tumor cell lines (So and Ouchi, 2010). For example, TLR3, TLR4, TLR7, and TLR9 have significantly higher expression in ESCA samples compared to normal tissues, which are associated with poor prognosis and lymph node metastasis (Sheyhidin et al., 2011).

Large-scale genomic and transcriptome analyses have suggested that less than 3% of the human genome encodes proteins and at least 75% are actively transcribed to non-coding RNA (ncRNA) (Djebali et al., 2012; Li and Liu, 2019). According to sequence length, ncRNA are generally divided into small ncRNAs with size < 200 nucleotides and long ncRNA (lncRNA) larger than 200 nucleotides in length (Han Li and Chen, 2015). A large number of functional studies have shown that lncRNAs play critical roles in a variety of cellular processes by regulating gene expression through diverse mechanisms at transcriptional, post-transcriptional and epigenetic levels (Rinn and Chang, 2012; Perry and Ulitsky, 2016; Koch, 2017). Furthermore, altered lncRNAs expression has been widely discovered in various cancers and have been used as novel biomarkers for cancer diagnosis and prognosis (Prensner and Chinnaiyan, 2011; Qiu et al., 2013; Zhou et al., 2018a; Bao et al., 2019; Sun et al., 2020). Increasing evidence indicated that lncRNA is emerging a key regulator of TLR signaling and innate immunity (Murphy and Medvedev, 2016; Zhang et al., 2020; Zhou et al., 2020), altered lncRNAs expression mediated via control of TLR signaling have been implicated in ESCA (Tang et al., 2015). However, a comprehensive analysis of lncRNAs changes induced by TLRs and their roles in diagnosis and prognosis in ESCA is lacking.

In the present study, we tried to investigate the precise relationship between lncRNA perturbations and TLRs dysfunction by constructing the lncRNA-TLRs co-expression network involved in ESCA. Through integrative analysis of transcriptome data and clinical data, we identified and validated a four-lncRNA signature induced by TLRs for improving outcome prediction of ESCA patients.

## Materials and Methods

### Clinical and Molecular Profiles Data of ESCA Patients

Clinical and lncRNA expression profile of 179 ESCA patients and paired 179 normal tissues profiled by Agilent-038314 CBC Homo sapiens lncRNA + mRNA microarray V2.0 were obtained from the Gene Expression Omnibus (GEO) database (the accession number is GSE53625)^1^ (Li et al., 2014). All 177 ESCA patients with survival information > 1 month were randomly divided into the discovery set (*n* = 120) for biomarker identification and the internal testing set for validation (*n* = 57) according to the ratio of 2:1 (Hartwell et al., 2018; Penn-Nicholson et al., 2019; Garrido et al., 2020). Clinical and RNA-seq data of another ESCA patient set (including 160 with survival information) was obtained from The Cancer Genome Atlas (TCGA) database^2^ for the independent validation. The clinical characteristics of ESCA patients in each dataset were shown in Table 1.

**TABLE 1:** Clinical characteristics of ESCA patients in each dataset.

Covariates	Discovery set (*n* = 120)	Internal testing set (*n* = 57)	TCGA set (*n* = 160)
Age, years (mean ± SD)	59.9 ± 12.1	57.8 ± 8.8	62.1 ± 11.9
**Gender, no (%)**			
Male	102 (85.0)	43 (75.4)	137 (85.6)
Female	18 (15.0)	14 (24.6)	23 (14.4)
**Stage, no (%)**			
I	6 (5.0)	3 (5.3)	16 (10.0)
II	48 (40.0)	28 (49.1)	68 (42.5)
III	66 (55.0)	26 (45.6)	49 (30.6)
IV	0 (0.0)	0 (0.0)	8 (5.0)
Unknown	0 (0.0)	0 (0.0)	19 (11.9)
**Alcohol use, no (%)**			
Yes	72 (0.6)	33 (57.9)	111 (69.4)
No	48 (0.4)	24 (42.1)	46 (28.7)
Unknown	0 (0.0)	0 (0.0)	3 (1.9)
**Pathological T, no (%)**			
T1	7 (5.8)	5 (8.8)	27 (16.9)
T2	14 (11.7)	11 (19.3)	37 (23.1)
T3	76 (63.3)	34 (59.6)	76 (47.5)
T4	23 (19.2)	7 (12.3)	4 (2.5)
Unknown	0 (0.0)	0 (0.0)	16 (10.0)
**Pathological *N*, no (%)**			
N0	54 (45.0)	28 (49.1)	65 (40.6)
N1	41 (34.2)	20 (35.1)	63 (39.4)
N2	18 (15.0)	4 (7.0)	9 (5.6)
N3	7 (5.8)	5 (8.8)	6 (3.8)
Unknown	0 (0.0)	0 (0.0)	26 (10.6)
**Vital status, no (%)**			
Alive	48 (40.0)	25 (43.9)	97 (60.6)
Dead	72 (60.0)	32 (56.1)	63 (39.4)

### TLR Genes

A total of 104 TLR-related genes were obtained from the TLR signaling pathways in Kyoto Encyclopedia of Genes and Genomes (KEGG) database.

### Processing and Analysis of lncRNA Expression Data

The lncRNA+mRNA array data were analyzed for data summarization, quantile normalization and quality control by using the GeneSpring software V11.5 (Agilent). Then probe sequence was aligned to lncRNA sequence of GENCODE using the blast software and obtained 6850 lncRNAs for further analysis. RNA-seq data of TCGA ESCA patients were re-annotated based on the lncRNA annotations in GENCODE, and obtained 15,873 lncRNAs.

Differential expression analysis for lncRNAs was performed between 179 paired ESCA and normal tissues using the R package “limma.” Those lncRNAs with | log2(fold change)| > 1 and false discovery rate (FDR) adjusted *p*-value < 0.05 were considered as significantly differentially expressed lncRNAs (Zhou et al., 2018a). Hierarchical clustering analysis was performed with the R package “pheatmap” using manhattan distance and “ward.D” method.

### Function Enrichment Analysis

Function enrichment analysis of mRNAs was performed with the R package “clusterProfiler” which can implement the statistical analysis and visualization of functional profiles for genes and gene clusters (Yu et al., 2012).

### Statistical Analysis

The relationship between expression levels of lncRNAs and overall survival (OS) were evaluated using the univariate and multivariate Cox regression analysis. LncRNA biomarkers were identified using the least absolute shrinkage and selection operator (LASSO) method. The lncRNAs-based signature was constructed using the linear sum of expression levels of lncRNAs biomarkers and the weights derived from multivariate Cox regression analysis (Zhou et al., 2018b; Zhou et al., 2018c). The optimal risk score was defined using the R package “maxstat.” Kaplan-Meier estimate and the log-rank test were used to compare survival differences between high-risk group and low-risk group. The prognostic value of signature was assessed using time-dependent receiver-operating characteristic (ROC) analysis. All statistical analyses were performed with R software (version 3.6.3).^3^

## Results

### Identification of TLR-Related lncRNAs in ESCA

**FIGURE 1:**
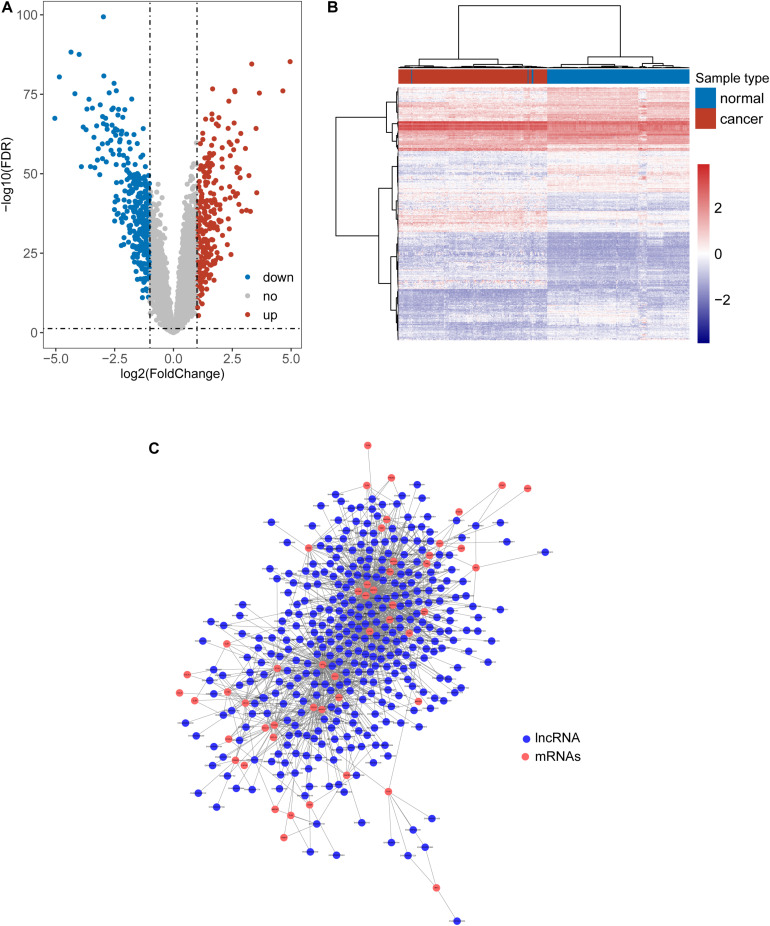
Identification of Toll-like receptors-induced lncRNAs in ESCA. **(A)** Volcano Plots of differentially expressed lncRNAs. **(B)** Heatmap of hierarchical clustering analysis based on differentially expressed lncRNAs. **(C)** The global view of a TLR-related lncRNAs-mRNA network.

We first tried to identify the lncRNAs associated with ESCA. Expression profiles of lncRNAs between 179 paired ESCA patients and normal tissues were compared to find differentially expressed lincRNAs from data of RNA-seq or microarray by performing analyses with the R software package bio-conductor “limma” (Zhou et al., 2018a). We identified a total of 587 lncRNAs with significant difference (| log2(fold change)| > 1 and FDR adjusted *p*-value < 0.05). Among them, 258 lncRNAs were found to be up-regulated and 329 lncRNAs to be down-regulated in ESCA (Figure 1A and Supplementary Table 1). Hierarchical clustering analysis suggested that the expression pattern of these 587 lncRNAs can significantly distinguish between ESCA patients and normal tissues (chi-square test *p* < 2.2e-16) as shown in Figure 1B. Then we examined the correlation between expression levels of 104 TLR-related genes and 587 differentially expressed lncRNAs by calculating the Pearson correlation coefficient. Finally, 357 lncRNAs were significantly correlated with that of at least one of TLR genes (Pearson correlation coefficient > 0.6 and *p* < 0.05) and were defined as TLR-related lncRNAs. As shown in Figure 1C, we constructed a TLR-related lncRNAs-mRNA network which included 1404 edges involved in 51 TLR genes and 357 lncRNAs.

### Identification of a Four-lncRNA Signature Induced by TLRs in the Discovery Set

**FIGURE 2:**
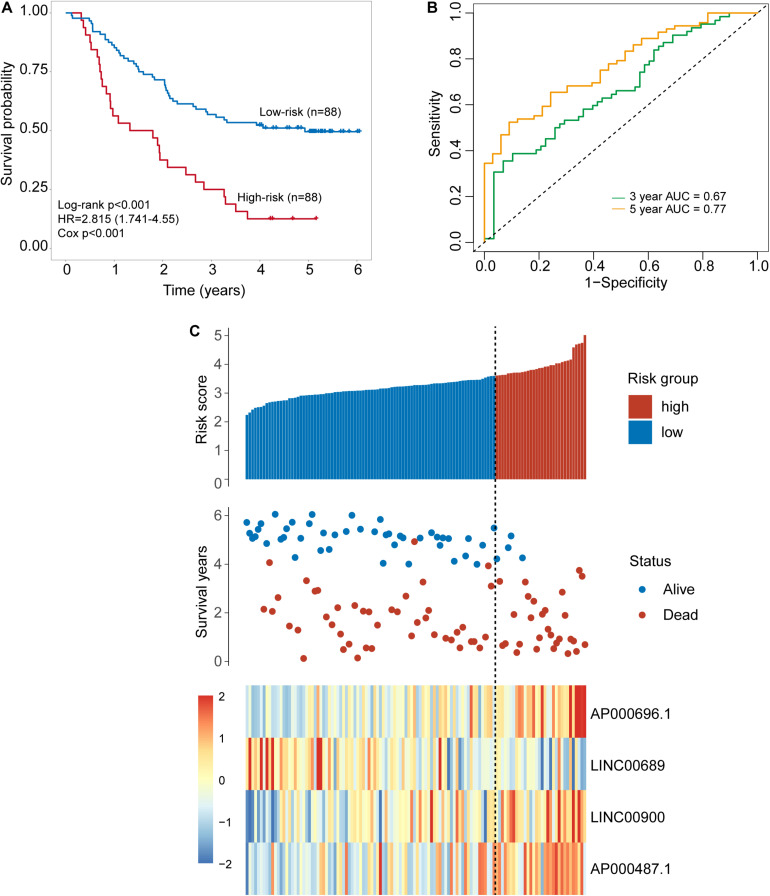
Development of a four-lncRNA signature induced by Toll-like receptors in the discovery set. **(A)** Kaplan–Meier survival curves of overall survival between the high-risk group and low-risk group stratified by four-lncRNAs signature. **(B)** The 3- and 5-year time-dependent ROC analysis. **(C)** Distribution of risk scores, patients’ survival status and lncRNAs expression pattern.

To identify potential TLR-related lncRNA biomarkers for predicting OS, we performed feature selection for 357 lncRNAs in the TLR-related lncRNAs-mRNA network in the discovery set, and identified four TLR-related lncRNAs (*AP000696.1*, *LINC00689*, *LINC00900*, and *AP000487.1*) as optimal biomarkers, which were significantly associated with OS of ESCA patients (Table 2). Then these four TLR-related lncRNAs biomarkers were integrated into a signature using the linear sum of expression levels of lncRNAs biomarkers and the weights derived from multivariate Cox regression analysis as follows: four TLR-related lncRNAs signature (four-TLR-lncRNA signature) = (0.239 × expression level of AP000696.1) + (−0.240 × expression level of LINC00689) + (0.124 × expression level of LINC00900) + (0.239 × expression level of AP000487.1). The optimal risk cutoff value of the four-TLR-lncRNA signature was determined using the R package “maxstat” in the discovery set. The optimal risk cutoff value of four-TLR-lncRNA could stratify 120 patients into the high-risk group (*n* = 88) and low-risk group (*n* = 32) with significantly different OS (Log-rank test *p* < 0.001) (Figure 2A). As shown in Figure 2A, the low-risk patients have a better OS (median 4.93 years) than those with high-risk (median 1.56 years). The five- survival rate of patients in the low-risk group is 49.5%, which is higher than those in the high-risk group (12.5%). The AUC for the four-TLR-lncRNA signature prognostic model was 0.77 at five years and 0.67 at three years of OS (Figure 2B). The distribution of risk scores, the survival status and lncRNA expression of patients were ranked by risk score and were shown in Figure 2C. As shown in Figure 2C, three lncRNAs (*AP000696.1*, *LINC00900*, and *AP000487.1*) are associated with high-risk and are up-regulated expressed in high-risk patients, and one lncRNAs (*LINC00689*) tended to be a protective factor and is up-regulated in the low-risk group.

**TABLE 2:** Four TLR-related lncRNA biomarkers significantly associated with the overall survival in the discovery patient set.

Ensembl id	Gene name	Genomic location	HR	95 % CI	*p*-value
ENSG00000231324	AP000696.1	Chr 21: 36,632,681–36,637,033 (−)	1.36	1.147–1.614	0.004
ENSG00000231419	LINC00689	Chr 7: 159,006,522–159,030,195 (+)	0.637	0.44–0.923	0.017
ENSG00000246100	LINC00900	Chr 11: 115,753,889–115,760,646 (−)	1.277	1.07–1.524	0.007
ENSG00000246889	AP000487.1	Chr 11: 70,372,246–70,398,488 (−)	1.039	1.039–1.633	0.022

### Validation of the Four-TLR-lncRNA Signature in the Internal Testing Set

**FIGURE 3:**
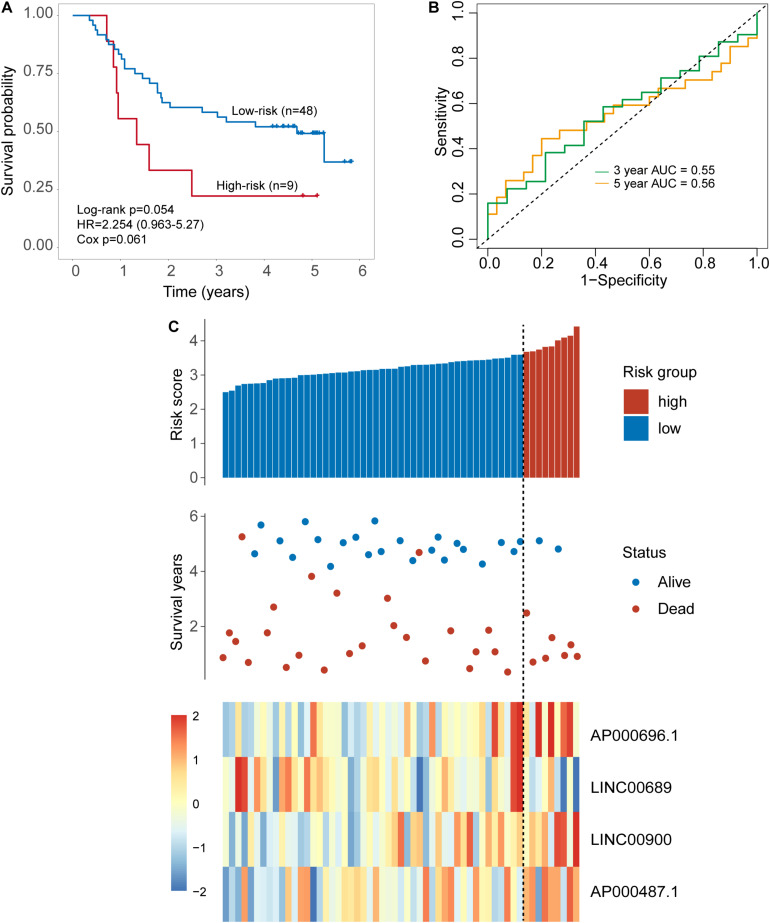
Validation of the TLR-induced four-lncRNA signature in the internal testing set. **(A)** Kaplan–Meier survival curves of overall survival between the high-risk group and low-risk group stratified by a four-lncRNAs signature. **(B)** The 3- and 5-year time-dependent ROC analysis. **(C)** Distribution of risk scores, patients’ survival status and lncRNAs expression pattern.

The same score formula and risk cutoff value obtained from the discovery set were applied to the patients in the internal testing set and calculate the risk score for each patient. With the four-TLR-lncRNA signature, patients of the internal testing set were classified into the high-risk group (*n* = 9) and low-risk group (*n* = 48). As shown in Figure 3A, the survival time of the high-risk group patients was significantly shorter than that of low-risk group patients (median survival 1.34 years vs. 4.69 years, log-rank test *p* = 0.054) (Figure 3A). The five-survival rate of patients in the low-risk group is 49.2%, whereas the corresponding five-survival rate of patients in the high-risk is 22.2%. The AUC for the four-TLR-lncRNA signature prognostic model was 0.56 at five years and 0.55 at three years of OS (Figure 3B). The distribution of risk scores, the survival status and lncRNA expression of patients were ranked by risk score and were shown in Figure 3C. As observed in the discovery set, the expression patterns of four TLR-related lncRNAs biomarkers are consistent with that in the discovery set. Three lncRNAs (*AP000696.1*, *LINC00900*, and *AP000487.1*) are risk factors, whereas the lncRNAs LINC00689 is a protective factor.

### Independent Validation of the Four-TLR-lncRNA Signature in the TCGA Set With Cross-Platform

**FIGURE 4:**
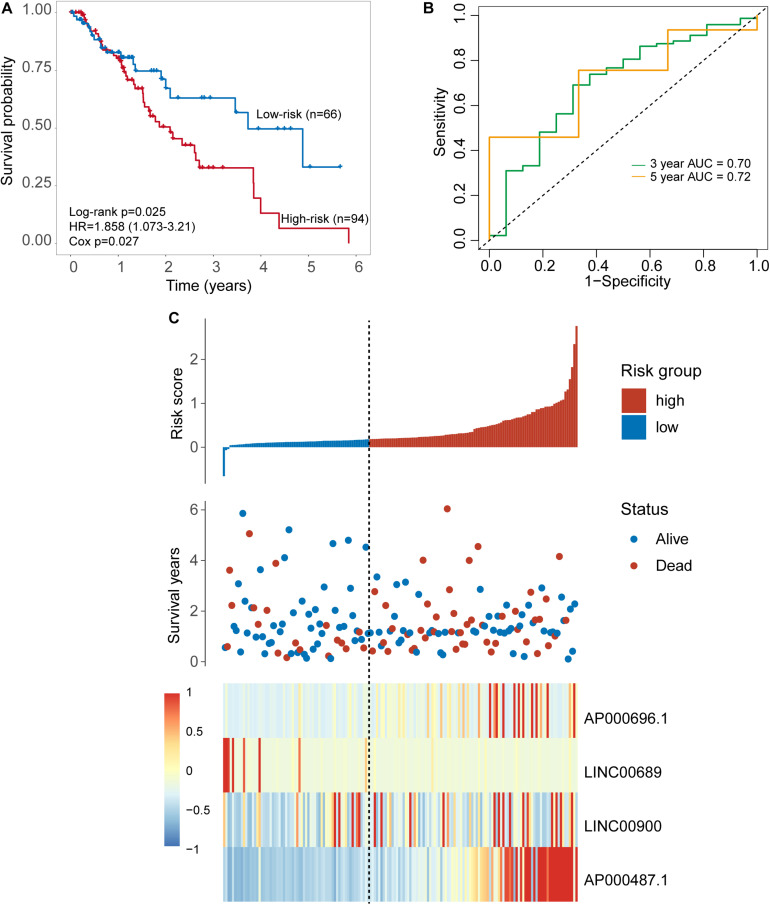
Further validation of the TLR-induced four -lncRNA signature in the TCGA set. **(A)** Kaplan–Meier survival curves of overall survival between the high-risk group and low-risk group stratified by four-lncRNAs signature. **(B)** The 3- and 5-year time-dependent ROC analysis. **(C)** Distribution of risk scores, patients’ survival status and lncRNAs expression pattern.

To further examine the robustness of the four-TLR-lncRNA signature in predicting OS, we tested the prognostic value of the four-TLR-lncRNA signature in another completely independent TCGA set with RNA-seq platform. When the four-TLR-lncRNA signature was applied to the TCGA set, the optimal risk cutoff value classified 160 patients into the high-risk group (*n* = 94) and low-risk group (*n* = 66). As shown in Figure 4A, there is a significant difference in OS between high-risk and low-risk groups. As in the discovery and internal testing sets, patients in the high-risk group had significantly shorter OS (median 2.09 years) than those in the low-risk group (median 3.73 years). The five-survival rate of patients in the low-risk group is 33.1%, whereas the corresponding five-survival rate of patients in the high-risk is 6.6%. The AUC for the four-TLR-lncRNA signature prognostic model was 0.72 at five years and 0.70 at three years of OS (Figure 4B). The distribution of risk scores, the survival status and lncRNA expression of patients were ranked by risk score and were shown in Figure 4C. As observed in the discovery and internal testing sets, the expression patterns of four TLR-related lncRNAs biomarkers are consistent with that in the discovery and internal testing sets. Three lncRNAs (AP000696.1, LINC00900 and AP000487.1) are risk factors, whereas the lncRNAs LINC00689 is a protective factor.

### Prognostic Value of the Four-TLR-lncRNA Signature Is Independent of Clinical Factors

We next performed the univariate and multivariate analysis with the four-TLR-lncRNA signature and other clinical factors (including age, gender, stage and alcohol) to examine whether the survival prediction ability of the four-TLR-lncRNA signature is independent of other clinical factors in three patient sets. In the discovery set, although univariate analysis revealed that the four-TLR-lncRNA signature (*p* < 0.001), age (*p* = 0.012), and gender (*p* = 0.07) were all significantly or marginally significantly associated with OS, the four-TLR-lncRNA signature (*p* < 0.001) and age (*p* = 0.003) were significant in the multivariate analysis. In the independent TCGA testing set, the four-TLR-lncRNA signature (*p* = 0.034) and stage (*p* = 0.017 and <0.001) were independent prognostic factors in the multivariate analysis (Table 3).

**TABLE 3:** Univariate and Multivariate Cox Regression Analysis of Overall Survival in each patient set.

Variables	Univariable Model			Multivariable Model		
	HR	95% CI of HR	*p*-value	HR	95% CI of HR	*p*-value
Discovery set (*n* = 120)						
**Risk group**						
High vs. Low	2.815	1.741–4.552	<0.001	3.089	1.900–5.023	<0.001
Age	1.036	1.008–1.066	0.012	1.045	1.015–1.075	0.003
**Stage**						
II vs. I	2.088	0.495–8.801	0.316			
III vs. I	3.122	0.756–12.895	0.116			
**Gender**						
Male vs. Female	0.582	0.324–1.046	0.070			
**Alcohol**						
Yes vs. No	0.790	0.496–1.258	0.321			
Internal testing set (*n* = 57)						
**Risk group**						
High vs. Low	2.254	0.963–5.275	0.061			
Age	1.009	0.97–1.05	0.656			
**Stage**						
II vs. I	Inf	0 – Inf	0.998			
III vs. I	Inf	0 – Inf	0.998			
**Gender**						
Male vs. Female	1.214	0.523–2.821	0.651			
**Alcohol**						
Yes vs. No	0.981	0.48–2.004	0.958			
TCGA testing set (*n* = 160)						
**Risk group**						
High vs. Low	1.858	1.073–3.217	0.027	1.998	1.054–3.786	0.034
Age	0.991	0.971–1.012	0.394			
**Stage**						
II vs. I	2.020	0.589–6.931	0.264	1.772	0.516 – 3.786	0.364
III vs. I	5.078	1.447–17.820	0.011	4.614	1.314 – 16.203	0.017
IV vs. I	14.918	3.734–59.601	< 0.001	11.837	2.936 – 47.712	< 0.001
**Gender**						
Male vs. Female	2.081	0.833–5.198	0.116			
**Alcohol**						
Yes vs. No	0.718	0.428–1.205	0.210			

### Functional Analysis of the Four-TLR-lncRNA Signature

**FIGURE 5:**
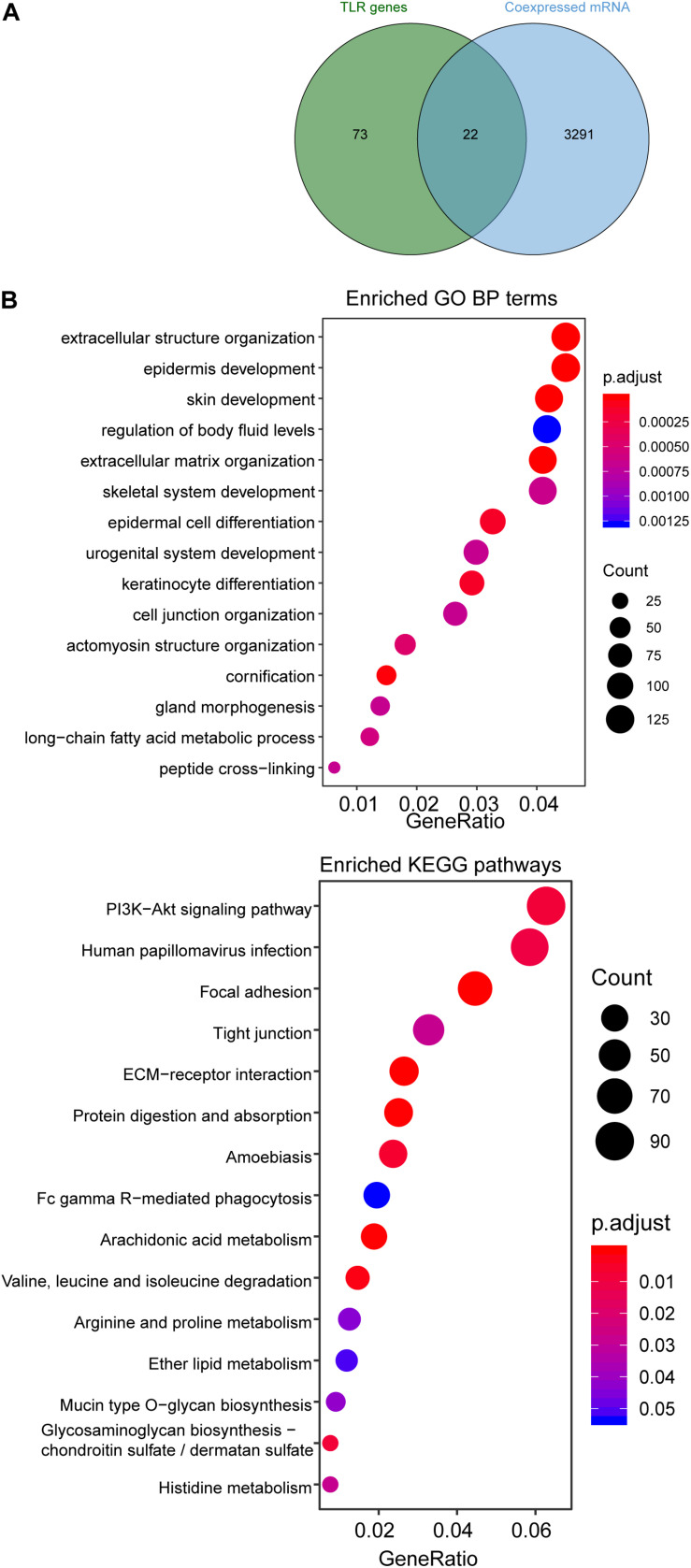
Functional enrichment analysis. **(A)** Venn diagram of co-expressed genes with lncRNAs and known TLR genes. **(B)** Enriched GO terms and KEGG pathways.

We first examined the correlation between expression levels of each of four TLR-related lncRNA biomarkers and mRNAs using the Pearson correlation coefficient and identified 3313 mRNAs related to lncRNA biomarkers. Of them, 22 mRNAs are well-known TLR genes. The results of the hypergeometric test revealed that TLR genes were marginally significantly enriched in mRNAs co-expressed with lncRNA biomarkers (hypergeometric test *p* = 0.076) (Figure 5A). We further performed functional enrichment analysis of GO and KEGG for 3313 mRNAs related to lncRNA biomarkers and selected top 15 enriched GO terms and KEGG pathways, which were shown in Figure 5B. We found that mRNAs co-expressed with lncRNA biomarkers are enriched in TLR-related and cancer-related GO terms and KEGG pathways such as ECM-receptor interaction, Focal adhesion and PI3K-Akt signaling pathway.

## Discussion

In addition to traditional treatments, including esophagectomy followed by chemotherapy and radiation therapy, other treatment options for ESCA patients continue to evolve, such as targeted drug therapy and immunotherapy (Wald et al., 2017). However, most ESCA patients are still faced with a poor prognosis with a five-year relative survival rate of about 20%. With considerable progress in our understanding of molecular characteristics of ESCA, it is now known that ESCA is a heterogeneous disease characterized by different molecular features associated with varied outcomes. Therefore, molecular profiles, including DNA, RNA, or proteins, have been proven to be a promising marker for improving clinical decision-making for diagnosis and prognosis of ESCA patients (Tanzer et al., 2013). LncRNAs have been found to be expressed in the cell and/or tissue/tumor-specific manner, highlighting their emerging roles as novel molecular markers in various cancers (Prensner and Chinnaiyan, 2011; Zhou et al., 2015a; Zhou et al., 2015b; Arun et al., 2018). Recent studies showed that lncRNAs appear to be a critical regulator in the immune system (Chen et al., 2017; Wang and Zheng, 2018). However, a comprehensive analysis of lncRNAs changes induced by TLRs and their roles in diagnosis and prognosis in ESCA is still in its infancy.

In this study, we first examined the expression pattern of lncRNAs between 179 paired ESCA patients and normal tissues and identified 587 differentially expressed lncRNAs, implying their potential roles in ESCA development. By investigating the co-expression relationship between dysregulated lncRNAs and known TLR genes, we found that 357 of 587 differentially expressed lncRNAs are significantly correlated with at least one of TLR genes, suggesting that dysregulated expression of these 357 lncRNAs may be induced by TLR genes. A global network between TLR gene and lncRNAs was constructed, which provides a potential way to understand the mechanisms by which lncRNAs regulate TLR-driven responses in the innate immune system for ESCA development. To further explore potential clinical implication of TLR-related lncRNAs in ESCA, we performed LASSO analysis for feature selection and identified four prognostic lncRNAs (*AP000696.1*, *LINC00689*, *LINC00900*, and *AP000487.1*) from the lists of 357 TLR-related lncRNAs. To accelerate the clinical application, these four TLR-related prognostic RNAs were integrated into a lncRNAs-based signature, which was capable of stratifying patients into high-risk and low-risk groups with significantly different OS in the discovery set. Further analysis in different independent patient sets also confirmed the robustness of the prognostic value of four-TLR-lncRNA signature in predicting OS of ESCA patients. Moreover, results of multivariate analysis in different patient sets indicated that the four-TLR-lncRNA signature is an independent factor after adjusted by other clinical factors (including age, gender, stage, and alcohol).

Of four lncRNAs biomarkers, *AP000696.1* is essential in the development of ectoderm and epithelial cells, and may sever as prognostic biomarker (Li et al., 2017). It has been reported that *LINC00900* is significantly up-regulated by all-trans-retinoic acid and down-regulated by vitamin D (Riege et al., 2017). LINC00689 has been observed to be deregulated expressed in ESCC. Furthermore, *LINC00689* also was recently reported to be involved in osteosarcoma progression via the miR-655/SOC18 axis (Xing et al., 2020) and growth, metastasis and glycolysis of glioma cells by targeting miR-338-3p/PKM2 axis (Liu et al., 2019). *In silico* functional analysis demonstrated that co-expressed genes with the four-TLR-lncRNA signature tended to be enriched in TLR-related and cancer-related biological progress and pathways. For example, damage-associated molecules patterns (DAMPs) have been found to be ligands for TLRs. Recent studies have reported that the extracellular matrix (ECM)-driven DAMPs contributed to the activation of TLR4 signaling during the tumor progression (Kelsh and McKeown-Longo, 2013). The iNOS/Src/Fak axis has revealed critical roles for macrophages in TLR-mediated cell motility (Maa et al., 2011). Coordinate regulation of TLR-mediated arachidonic acid metabolism in macrophages has been reported to be involved in a variety of innate immune responses (Ruiperez et al., 2009). Although *in silico* functional analysis revealed the functional implication of the four-TLR-lncRNA signature in TLR pathways, further experimental studies were needed for verifying and deciphering regulatory mechanisms between these identified lncRNAs and TLR pathways in ESCA. Another limitation is that the prognostic value of the four-TLR-lncRNA signature was analyzed only in public datasets of ESCA, therefore, further retrospective studies or prospective clinical trials are needed.

In conclusion, in this study, we investigated functional roles of lncRNAs in TLR signaling pathways and their effects on the outcome of ESCA patients. The four-lncRNA signature induced by TLRs identified in this study represents a promising biomarker for outcome prediction and provides new candidate targets for therapeutic intervention via targeting lncRNAs and their TLR partners in ESCA patients.

## Data Availability Statement

Publicly available datasets were analyzed in this study. This data can be found here: Clinical information of ESCA patients was downloaded from GSE53625 (https://www.ncbi.nlm.nih.gov/geo/query/acc.cgi?acc=GSE53625) and TCGA (https://www.cancer.gov/).

## Author Contributions

DZ and XW conceived and designed the experiments. JL, YW, YC, and RX performed the experiments and analyzed the data. JL and YW wrote the manuscript. All authors read and approved the final manuscript.

## Conflict of Interest

The authors declare that the research was conducted in the absence of any commercial or financial relationships that could be construed as a potential conflict of interest. The reviewer XC declared a shared affiliation with the authors to the handling editor at time of review.

## Footnotes

1 https://www.ncbi.nlm.nih.gov/geo/query/acc.cgi?acc=GSE53625

2 https://www.cancer.gov/

3 www.r-project.org

## Supplementary Material

TABLE S1 Lists of differentially expressed lncRNAs between 179 paired ESCA patients and normal tissues.
